# Synergistic Effect of *Rhodiola rosea* and Caffeine Supplementation on the Improvement of Muscle Strength and Muscular Endurance: A Pilot Study for Rats, Resistance Exercise-Untrained and -Trained Volunteers

**DOI:** 10.3390/nu15030582

**Published:** 2023-01-22

**Authors:** Chang Liu, Haotian Zhao, Yi Yan, Weijun Yang, Songyue Chen, Ge Song, Xuehan Li, Yujia Gu, Hezhang Yun, Yi Li

**Affiliations:** 1 School of Sport Science, Beijing Sport University, Beijing 100084, China; 2 Department of Physical Education, Jiangnan University, Wuxi 214122, China; 3 School of Pharmaceutical Sciences, Peking University, Beijing 100191, China; 4 The Public Sports Department of the School, Zhejiang Guangsha Vocational and Technical University of Construction, Dongyang 321000, China; 5 State Key Laboratory of Toxicology and Medical Countermeasures, Beijing Institute of Pharmacology and Toxicology, Beijing 100850, China

**Keywords:** *Rhodiola rosea*, Caffeine, erythropoietin, dopamine, physical performance

## Abstract

Multi-level studies have shown that *Rhodiola rosea* (RHO) and Caffeine (CAF) have the potential to be nutritional supplements to enhance physical performance in resistance exercise-untrained and -trained subjects. This study examined the synergistic effects of RHO (262.7 mg/kg for rats and 2.4 g for volunteers) and CAF (19.7 mg/kg for rats and 3 mg/kg for volunteers) supplementation on improving physical performance in rats, resistance exercise-untrained volunteers and resistance exercise-trained volunteers. Rats and volunteers were randomly grouped into placebo, CAF, RHO and CAF+RHO and administered accordingly with the nutrients during the training procedure, and pre- and post-measures were collected. We found that RHO+CAF was effective in improving forelimb grip strength (13.75%), erythropoietin (23.85%), dopamine (12.65%) and oxygen consumption rate (9.29%) in the rat model. Furthermore, the current results also indicated that the combination of RHO+CAF significantly increased the bench press one-repetition maximum (1RM) (16.59%), deep squat 1RM (15.75%), maximum voluntary isometric contraction (MVIC) (14.72%) and maximum repetitions of 60% 1RM bench press (22.15%) in resistance exercise-untrained volunteers. Additionally, despite the excellent base level of the resistance exercise-trained volunteers, their deep squat 1RM and MVIC increased substantially through the synergistic effect of RHO and CAF. In conclusion, combined supplementation of RHO+CAF is more beneficial in improving the resistance exercise performance for both resistance exercise-untrained and -trained volunteers. The present results provide practical evidence that the synergies of RHO and CAF could serve as potential supplementary for individuals, especially resistance exercise-trained subjects, to ameliorate their physical performances effectively and safely.

## 1. Introduction

*Rhodiola rosea* (RHO) is a typical traditional herbal medicine widely used in Russia and Scandinavian and Asian countries (e.g., China and India) for its various health-promoting effects [1]. Its main ingredient, rhodioloside (also known as salidroside), seems to be effective in reducing fatigue and enhancing athletic performance [1,2]. Studies have shown that rhodioloside achieves its functions by modulating the hypoxia-inducible factor-1 signaling pathway, which is a heterodimeric transcription factor expressed in numerous body tissues and is responsible for the induction of erythropoietin (EPO) gene expression under hypoxic conditions [3,4]. The newly generated EPO can bind to the EPO receptor, which is highly expressed in the bone marrow hematopoietic stem cells, via body circulation. It then promotes the activation of the JAK2-STAT5 signaling pathway to produce early erythrocytes, which subsequently develop into mature erythrocytes [5,6]. Recent studies have also shown that RHO supplementation can enhance explosive resistance training performance, especially the bench press 1RM, while also increasing mean bench press velocity but decreasing bench press repetition volume [7,8]. Four weeks’ intake of RHO improves endurance exercise performance but did not alter other parameters, including muscle strength, limb movement speed, reaction time, and attention, while also increasing the VO_2peak_ [9].

Caffeine (1,3,7-Trimethylpurine-2,6-dione, CAF) is the main ingredient in coffee that effectively promotes athletic capacities [10,11,12], such as anaerobic exercise capacity, strength and muscular and aerobic endurance [13,14,15]. As one of the most well-known and widely used adenosine receptor antagonists, CAF is effective in promoting the release of neurotransmitters such as dopamine and catecholamine (epinephrine and norepinephrine) into action, which further improves exercise performance [16,17]. In vitro evidence indicates that CAF in relatively high concentrations can also increase skeletal muscle strength and muscle power, which can further enhance the above-mentioned ability of adenosine receptors to reinforce resistance exercise [18,19].

CAF was previously identified as a banned substance in sports and was banned between 1984 and 2004 because it was shown to enhance athletic performance in sports such as running, swimming and cycling [20]. The World Anti-Doping Agency decided to remove CAF from the Prohibited List and lifted the ban as of 1 January 2004, despite growing evidence that it is a sports booster. As a result, ordinary people and athletes alike are consciously or unconsciously taking large amounts of caffeine to enhance their athletic performance [21]. For example, 75–90% of high-level athletes consume CAF as a nutritional supplement before or during competition, especially weightlifters [15,21].

It has been reported that athletes typically receive 3–6 mg/kg doses of CAF to acutely enhance resistance exercise performance [22,23] and that 3 mg/kg CAF supplementation was as effective in enhancing resistance exercise performance as 6 mg/kg CAF supplementation [12]. However, a CAF dose above 9 mg/kg often fails to elicit a further positive response to various performance enhancements, and may also lead to a series of side effects associated with excessive CAF intake, such as tachycardia, headache and anxiety [24,25,26]. The long-term intake of CAF leads to an upregulation of adenosine receptor expression, resulting in a progressive reduction in the stimulatory effect on adenosine receptors [27,28]. For example, the effects of CAF resistance on the human body have gradually occurred after 28 days of long-term CAF intake [29,30]. Furthermore, among habitual CAF users, acute CAF intake even fails to improve their athletic performance [15]. On the other hand, there is still some evidence that regular caffeine intake does not moderate acute ergogenic benefits of caffeine intake in sports performance [31].

**Figure 1:**
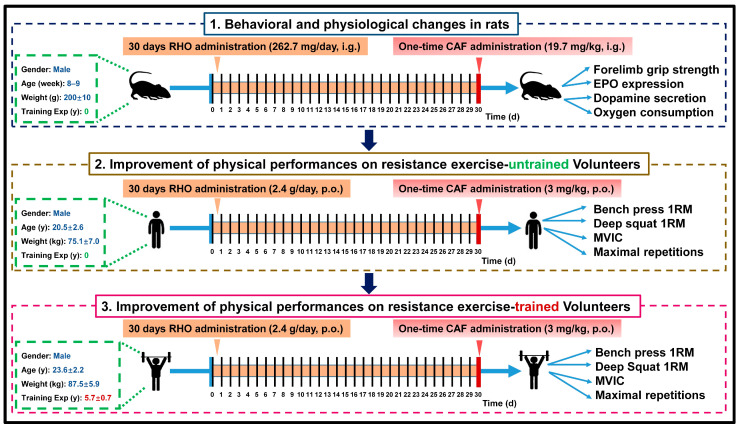
Schematic diagram of grouping, administration and experimental approaches. (1) Male SD rats were treated with 262.7 mg/kg *Rhodiola rosea* for 30 consecutive days with training and 19.7 mg/kg CAF on the last day, followed by measurement of relevant parameters (n = 12 × 4 groups = 48 samples). (2) Volunteers without resistance training experience committed to taking *Rhodiola rosea* for 30 days while participating in the training program. When the 30-day period ended, they took a single dose of 3 mg/kg CAF and then measured the relevant parameters (n = 12 × 4 groups = 48 samples). (3) Resistance exercise-trained volunteers with 5.7 ± 0.7-year of resistance training experience took *Rhodiola rosea* for 30 days during their participation in the training program. When the 30 days ended, they took a one-time dose of 3 mg/kg CAF, followed by the measurement of the relevant indicators (n = 12 × 2 groups = 24 samples).

Therefore, in this study, we hypothesized that the combined supplementation of RHO and CAF could further improve muscle strength and muscular endurance compared to supplementation with RHO or CAF alone because of the superimposed effects of the two supplements due to their different mechanisms of application. Accordingly, as schematized in Figure 1, we first verified in the rat models whether the corresponding doses of RHO+CAF could improve forelimb grip strength, EPO level and dopamine level. It has been shown that both RHO and CAF can help increase the level of oxidative phosphorylation in mitochondria to further promote muscle exercise, so the oxygen consumption rate affected by the synergistic effect of RHO and CAF was also evaluated here. Subsequently, age-matched participants without habitual CAF consumption (including resistance exercise-untrained and -trained volunteers) were selected as the primary respondents. The current work was conducted to investigate whether an acute single dose of 3 mg/kg CAF after 2.4 g daily intake of RHO for 30 days with training can effectively improve the resistance exercise performance, including the administration of nutritional supplements in volunteers who barely have any resistance training experience, and in male volunteers with approximately 5 years of resistance training experience. The main metrics assessed in resistance exercise-untrained and -trained volunteers after administration of different supplementations included bench press 1RM, deep squat 1RM, MVIC and maximal repetitions of 60% 1RM bench press.

## 2. Materials and Methods

### 2.1. Animals

Male Sprague–Dawley rats (age: 8–9 weeks, weight: 200 ± 10 g) were provided by the Department of Laboratory Animal Science, Peking University (Beijing, China), and housed in specific pathogen-free (SPF) rooms according to production certificate No. SCXK (Beijing) 2016-0010, and use license No. SYXK (Beijing) 2016-0041. Two rats were housed per cage, and the SPF rooms were controlled at 25 ± 1 °C, with the relative air humidity controlled at 50–60% and a 12-hour light/dark cycle. All animal procedures were in accordance with the Regulations for the Administration of Laboratory Animals and were approved by the Ethics Committee of Beijing Sport University (Ethics Committee No. 2022055A).

Rats were randomly divided into four groups (12 rats in each group) and treated intragastrically (i.g.) by gavage. CAF capsules were purchased from Nutricost, UT, USA, containing 200 mg of caffeine anhydrous for each capsule, and the pharmaceutical excipients were rice flour and gelatin (capsule), while the recommended concentration for improving physical resistance in humans was 3 mg/kg. RHO was obtained from Beijing Tong Ren Tang Pharmaceuticals, China, and the recommended dose was 2.4 g per day, containing 12 mg rhodioloside in total, and the pharmaceutical excipients are starch and gelatin (capsule). To facilitate administration, the powders from the CAF and RHO capsules were removed and dissolved in water to produce a highly concentrated solution (rather than a mixed suspension) and administrated immediately after the solution had been prepared. Based on the dose–conversion relationship between animals and humans, we administered 262.7 mg/kg of RHO and 19.7 mg/kg of CAF to the male SD rats [32].

The specific experimental procedure is shown in Figure 1. In the placebo group, neither RHO nor CAF was administered throughout the whole process, while in the RHO group, 262.7 mg/kg of RHO was given by gavage for 30 days. In the CAF group, 19.7 mg/kg of CAF was given by gavage on the last day half an hour before the test, while in the RHO+CAF group, 262.7 mg/kg of RHO was administered by gavage for 30 days, and 19.7 mg/kg of CAF was administered by gavage on the last day.

### 2.2. Resistance Training Protocol for Rats

The 30-day resistance training protocol was modified from a previous study [33]. Briefly, the SD rats from different groups were familiarized with the custom-made vertical ladder (height × width: 90 cm × 15 cm) three times a week before the study. On the first day, the rats climbed without any additional load. For the next two days, a bag of lead weights equivalent to 50% of the rat’s body weight was attached to the proximal portion of the tail using double-sided tape. All the rats then began gradual resistance training three times per week. The first load was 75% of the rat’s body weight, and thereafter, the load was increased by 30 g on each climb until the rat could no longer reach the top of the ladder. The subsequent training sessions consisted of nine trials. For the first three climbs, 50%, 75% and 90% of the previous maximum load were used. The load was then increased by 30 g until a new maximum load was reached. This new maximum load was then used for three attempts. Between climbing trials, the rats were allowed to rest for 90 s in the open chamber at the top of the ladder. The rats were neither punished nor rewarded to motivate them to climb; only an occasional gentle push on the rat’s back was used to initiate the climbing.

### 2.3. Forelimb Grip Strength Test

The grip strength of the front paws was measured using a Grip Strength Meter (Bileadbiosci Co., Ltd., Shenzhen, China). The grip strength was used to determine the maximum peak force generated by the rat pulled out the metal rod. The machine was mounted on a stable and firm table, while the rat was allowed to grasp the metal rod with its front paws while its tail was pulled backward on a horizontal plane. The peak tension, the force exerted on the metal rod before it lost its grip, was recorded in grams. The rat was pulled three times and the device recorded the highest peak tension. The body weight of each rat was measured and the results were presented as grip strength (g) per body weight (100 g), comparing the grip strength of placebo, RHO, CAF and RHO+CAF groups [34].

### 2.4. Analysis of EPO Gene Expression in Rat Kidney

Total ribonucleic acid (RNA) was extracted using Trizol Reagent (Invitrogen^TM^, Carlsbad, CA, USA) according to the manufacturer’s protocol. After collecting the blood, rats were rapidly decapitated. Approximately 10 mg of renal cortical tissue was thawed and homogenized in 1 mL of Trizol reagent. The concentration of RNA was quantified using a Nanodrop Spectrophotometer (NanoDrop ND-1000, Thermo Fisher Scientific Inc., Waltham, MA, USA). In total, 1000 ng of RNA was reverse-transcribed into cDNA using a High-Capacity cDNA Reverse Transcription Kit (Thermo Fisher Scientific Inc., Waltham, MA, USA). The primer specific sequences were as follows: the forward (5′-3′) of EPO was TACGTAGCCTCACTTCACTGCTT; the reverse (3′-5′) of EPO was GCAGAAAGTATCGTGTGAGTGTTC; the forward (5′-3′) of β-actin was CTTTCTACAATGAGCTGCGTG; the reverse (3′-5′) of β-actin was TCATGAGGTAGTCTGTCAGG. A RT-qPCR kit (Thermo Fisher Scientific, Waltham, MA, USA) and a StepOne Plus Real-time PCR system (Thermo Fisher Scientific, Waltham, MA, USA) were used to perform the quantitative real-time polymerase chain reaction (qPCR). Reactions were preheated at 95 °C for 10 min, followed by 40 cycles at 95 °C for 20 s, 60 °C for 20 s and 72 °C for 20 s. The qPCR data were quantified based on the number of cycles required to reach a specific detection threshold (Ct value) for the fluorescence generated by the amplification. Relative gene expression was quantified based on equal amounts of RNA (1 μg) and the average Ct value of each gene. Delta Ct (ΔCt = Ct_target gene_ − Ct_reference gene_) was calculated using the Ct values of genes in the same sample. β-actin was used as an endogenous control gene. ΔΔCt values were calculated using the following equation: ΔΔCt = (ΔCt_treated_ − ΔCt_untreated_). The normalized expression changes were expressed as 2^−ΔΔCt^ (β-actin control set to 100) [35].

### 2.5. Serum Erythropoietin (EPO) Analysis by ELISA

At the end of the forelimb grip strength tests, rats were anesthetized by intraperitoneal injection of sodium pentobarbital (50 mg/kg). Blood samples were collected from the right jugular vein on the last day after administration, and the rats were kept under anesthesia throughout the experiment. Serum samples were obtained by centrifugation at 12,000 rpm for 15 min after allowing the blood samples to clot at room temperature for 30 min.

The serum EPO levels were determined by an ELISA method using the standard EPO ELISA Kits (Cusabio Biotech Co., Ltd., Wuhan, China). This assay employed a quantitative sandwich enzyme immunoassay technique in which EPO in the samples or standards were sandwiched between pre-coated EPO antibody and biotin-conjugated EPO antibody. The Ab-Ag-Ab complex was labeled with horseradish peroxidase (HRP). After washing to remove any unbound reagents, the 3,3′,5,5′-tetramethylbenzidine substrate solution was added to the wells and color development was performed in proportion to the amount of EPO bound in the initial step. Color development was stopped before the intensity of the color was measured at 450 nm using a microplate reader. The serum samples were collected and stored at −80 °C until analysis, and we strictly followed the protocol suggested in the instructions [36].

### 2.6. Striatal Dopamine Analysis by ELISA

As the rats had already been decapitated, we separated the striatum from the brain. The whole process was carried out using the Rat Dopamine ELISA Kit (Cusabio Biotech Co., Ltd., Wuhan, China) in strict accordance with the manufacturer’s instructions. Briefly, 30 mg of the striatum was rinsed in PBS and stored overnight at −20 °C before homogenization in 300 μL of PBS. Two freeze–thaw cycles were performed to break the cell membrane. The homogenate was centrifuged at 20,000× *g* for 10 min at 4 °C. The supernatant was removed and immediately assayed. The allocated antigen standards and samples were added to each well of a 96-well plate pre-coated with primary antibodies. After adding biotin conjugate reagent and enzyme conjugate reagent to each well, the plate was incubated at 37 °C for 60 min. The plates were then rinsed 5 times with distilled water. Within 30 min of the color development reaction, absorbance was measured at 450 nm using a microplate reader [37].

### 2.7. Oxygen Consumption Rate Measurement of Muscle Fibers

Bilateral gastrocnemius muscles were isolated from the anesthetized rats after the strength testing as previously described [38]. The harvested muscle fibers were preserved in buffer (pH 7.2) consisting of 120 mM NaCl, 3.5 mM KCl, 1.3 mM CaCl_2_, 0.4 mM KH_2_PO_4_, 1 mM MgCl_2_, 5 mM HEPES supplemented with 2.5 mM D-glucose and 0.5 mM L-carnitine. Before analysis, the fibers were placed in an unbuffered, humidified incubator at 37 °C for 2 h for pre-equilibration.

Bioenergetics analyses were performed utilizing a Seahorse XFe24 Flux Analyzer as previously described with modifications [39,40]. The stock substrate of 500 mM pyruvate and inhibitors of 1 mg/mL Oligomycin, 1 mM Carbonyl cyanide-p-trifluoromethoxyphenylhydrazone (FCCP) and 1 mM Antimycin were prepared and adjusted to pH 7.2. The appropriate inhibitors and/or substrates (10× working concentrations made from stock) were sequentially loaded into the sensor cartridge ports and injected into the assay plate to reach the final concentration (1×) of the compound in each well. The amount of substrate loaded in each port is based on the initial volume of 500 μL buffer in the plate loaded with muscle fibers as follows: 50 μL (Oligomycin), 55 μL (FCCP+pyruvate) and 60 μL (Antimycin). The loaded reagents were sequentially injected through the ports of the calibration cartridge to final concentrations of 1 μg/mL Oligomycin, 400 nM FCCP + 10 mM pyruvate and 1 μM Antimycin.

Basal oxygen consumption rates (OCR, pMoles/min) of the pre-equilibrated muscle fibers were recorded using a loop of 4 min mixing, 3 min waiting and 3 min measurement (3 loops) before injection of Oligomycin to inhibit the ATP synthase (Phase(Oligo)). Three more measurement loops were recorded prior to the injection of FCCP plus pyruvate substrate to induce maximal oxygen consumption (Phase(FCCP/pyr)). Following the recording of 3 more measurement loops, Antimycin was injected for inhibiting the mitochondrial respiration to evaluate non-mitochondrial OCR (Phase(Anti)). Two measurement loops were recorded after Antimycin injection, and then the experiment was terminated. In this study, we focused more on the Phase(FCCP/pyr) since this parameter is an indicator of maximal mitochondrial function, which reflects the maximal aerobic endurance and strength of muscles under exhaustive exercise conditions.

### 2.8. Participants

This study was a randomized, double-blind, crossover clinical trial conducted at the Beijing Sport University and Peking University. Four groups of resistance exercise-untrained volunteers and two groups of resistance exercise-trained volunteers of similar age and BMI were recruited as participants from these two universities, and their somatotype profile is shown in Table 1.

The recruitment and screening process for the young male resistance exercise-untrained volunteers began 3 months prior to the formal human study with an initial measurement screen conducted to select suitable subjects from a large number of potential subjects. The volunteers (including placebo, CAF, RHO, RHO+CAF groups) who were eventually recruited for resistance exercise-training initially bench pressed approximately 25.21 ± 3.10, 24.79 ± 3.45, 24.58 ± 3.34, 25.00 ± 3.02 kg at one-repetition maximum, deep squatted approximately 36.25 ± 4.20, 36.04 ± 3.76, 35.83 ± 4.17, 35.63 ± 4.28 kg at one-repetition maximum, with MVICs of 179.17 ± 14.50, 180.25 ± 13.43, 177.42 ± 14.29, 178.67 ± 13.13 N·m and maximal repetitions of 60% 1RM bench press of 10.42 ± 1.08, 10.25 ± 0.97, 10.33 ± 1.07, 10.17 ± 1.11 times. These young male volunteers were primarily general college students who did not yet have experience with resistance exercise training. These forty-eight healthy, resistance exercise-untrained volunteers who willingly participated in the study were randomly divided into four groups (n = 12) to take the corresponding treatment while training, numbered 1–4: (1) placebo, (2) CAF, (3) RHO and (4) RHO+CAF.

Similarly, the recruitment process of the resistance exercise-trained volunteers started three months before the official human experiment. These resistance exercise-trained volunteers were mainly from Beijing Sport University, and they all had more than 5 years of resistance training experience; although they had more than three different sports-specific skills, they all had fitness and bodybuilding-specific skills, so their daily training process included not only aerobic training but also anaerobic training. These resistance exercise-trained volunteers had an initial state bench press 1RM of approximately 139.58 ± 3.17 kg (placebo) and 139.79 ± 3.61 kg (CAF+RHO), while the initial deep squat 1RM values were 192.08 ± 3.82 kg (placebo) and 191.67 ± 3.59 kg (CAF+RHO). Meanwhile, the initial MVIC values were 684.08 ± 10.85 kg (placebo) and 682.58 ± 11.33 kg (CAF+RHO), while the maximal repetitions of 60% 1RM bench press values were 17.92 ± 1.16 kg (placebo) and 18.17 ± 0.83 kg (CAF+RHO), all of which indicated a suitable strength training base and athletic performance. Due to the low number of resistance exercise-trained volunteers, we were only able to recruit 24 of them. These healthy volunteers with more than 5 years of resistance training experience were divided into 2 groups (n = 12) while training, and the corresponding treatments were numbered 5–6: (5) placebo and (6) RHO + CAF.

In order to maintain the scientific integrity of our study, and in accordance with the relevant experimental ethical requirements of the Beijing Sport University, we reached agreements and signed commitments with the young male resistance exercise-untrained volunteers and resistance exercise-trained volunteers three months prior to the formal conduct of human experiments that they would not be self-administered CAF and RHO until all the experiments were completed. In addition, subjects were repeatedly and periodically reminded throughout the study not to use CAF and RHO autonomously to ensure that the experimental process was free of unnecessary interference.

The specific experimental procedure is shown in Figure 1. All the participants had stated that they had not consumed any caffeine, tea, additional nutritional supplements, ergogenic aids or alcohol-containing products that could significantly affect their muscular performance spanning 3 months before this study, and they were also non-smokers. Additionally, all the participants had no neuromuscular or musculoskeletal disorders, and they were able to perform the bench press and the deep squat exercises. The group of participants with zero resistance training experience had not participated in any upper body exercises, including the bench presses, or lower body exercises, including the deep squats, before the study [41]. CAF capsules were purchased from Nutricost, UT, USA, containing 200 mg caffeine anhydrous for each capsule, and the pharmaceutical excipients were rice flour and gelatin (capsule). The CAF drug powder from the capsules was accurately weighed to the desired amount and then taken by the participants at a dose of 3 mg/kg. RHO was purchased from Beijing Tong Ren Tang Pharmaceuticals, Beijing, China, and was administrated as the recommended dose (2.4 g daily, containing 12 mg rhodioloside in total, and the pharmaceutical excipients are starch and gelatin (capsule)) for 30 days. RHO should be taken early in the day because it can disrupt sleep or produce vivid dreams (not nightmares), and it is contraindicative in heated conditions. RHO should not be used in patients with bipolar disorder who are prone to becoming manic when taking antidepressants or stimulants because of its activating antidepressant effect [42]. Therefore, when we recruited the participants, we avoided recruiting those who were taking antidepressants or stimulants, and we asked all the participants to take RHO on an empty stomach 30 min before breakfast and lunch. For the control group, we removed the drug powder from the CAF or RHO capsules. The participants were to intake the empty capsules (without RHO or CAF inside while indistinguishable from the other supplements given) without being informed [43].

It is important to note that in order to avoid the impacts of the placebo effect, the placebo group also drank the same amount of water when taking empty CAF and RHO capsules. Similarly, for the CAF single dosing group, they also needed to take empty RHO capsules, and for the RHO single dosing group, they were also required to take empty CAF capsules to avoid the placebo effect.

Participants did not attend any other strength training, use any other nutritional supplements, participate in any other relevant scientific research projects or have any health problems during the study period. Moreover, participants were fully informed of the study procedures, including any risks of participation in the study, before providing the written informed agreement. The experimental methods were conducted in accordance with the Declaration of Helsinki and were approved by the Human Research Ethics Committee of the Beijing Sport University (Ethics Committee No. 2022053H).

### 2.9. Training Protocol for Humans

The training program lasted 30 days and consisted of thirteen training sessions of the bench press and deep squat exercise. The training program was supervised by a number of professional trainers. Each of these trainers was responsible for supervising 3 subjects. The training exercises were performed on the same machine employed for the testing. For the first 15 days, participants performed 4 sets of 10 repetitions at 60% of their pre-training 1RM, and during the last 15 days, the training protocol was changed to 4 sets of 8 repetitions at 70% of the pre-training 1RM. The training program was designed to maximize the increases in muscle strength/muscle endurance for intermediate loads. Before the onset of the training protocol, participants received a plastic container with 30 unidentifiable capsules according to the group to which they were allocated [44]. It is worth noting that we only measured physical performance for bench press 1RM, deep squat 1RM, MVIC and maximal repetitions of 60% 1RM bench press 10 h after the last workout.

### 2.10. Daily Recordings of Dietary Intake

All participants, including resistance exercise-untrained and -trained volunteers, were instructed to maintain their habitual diets throughout the study. To further control habitual dietary intake, several packages were set up for participants to choose from, all of which met the appropriate national food safety requirements. Additionally, participants received written and verbal instructions to record the types and portion sizes of daily foods consumed everyday with the help of trained interviewers (nutritionists). The calculation of total energy intake per day was based on the National Nutrient Database for Standard Reference from the United States Department of Agriculture. Dietary intake was calculated as energy, in calories per day, and carbohydrates, protein and fat in grams per day [45]. Neither CAF nor RHO affected the participants’ appetite or food intake [46,47].

### 2.11. Bench Press/Deep Squat Max. Strength (Bench Press/Deep Squat 1RM)

The maximum weight of a single bench press or deep squat is a classic measure of absolute strength and is known as the 1RM (one-repetition maximum) [48]. The 1RM test is important for assessing baseline changes in a participant’s strength level and is an important indicator of strength setting in muscle training [49].

The bench press/deep squat 1RM test consists of three main steps. (1) The participant is warmed up with a light load (10–15 reps that can be easily performed) under professional instruction, which is adjusted by the professional trainer according to the participant’s personal specifications. The load for the bench press is approximately 25 ± 2.5 kg and for the deep squat is approximately 35 ± 2.5 kg. (2) Increase the load by 10–15%, so that the participant is able to complete about 6–8 repetitions (warm-up lift) with 2–5 min rest. After a further increase of 5–10% load, the participant will be able to perform 3–4 repetitions (conservative lift, no exertion). (3) Next, after a 2–5 min break, increase the load by 5–10% as in step 2, so that the participant can perform 1 or 2 repetitions. If the participant completes this, then rest for 2–5 min, and continue to increase the load by 5–10% for the bench press/deep squat 1RM. If the participant fails, then rest for 2–5 min, and subsequently reduce the load by a small amount before performing the final attempt. The overall bench press/deep squat 1RM measurement should be completed within a total of five attempts [50].

### 2.12. MVIC (Maximum Voluntary Isometric Contraction) Test for the Quadriceps

In this study, an American Isometric Force Testing System (Biodex Medical Systems Inc., Shirley, NY, USA) was used to measure the knee extension moment of the knee extension muscles of the participant’s legs, with a sampling frequency of 2000 Hz and a moment accuracy of ±1%. The test angle of the knee joint was set to 75°, and the test time, test frequency and interval time were also set correspondingly. The maximum knee extension torque test took 5 s per test and each test was repeated 3 times [51].

### 2.13. Maximal Repetitions of 60% 1RM Bench Press

Maximal repetitions of 60% 1RM bench press is a classic method for testing isotonic muscular endurance in the upper limb by testing the number of movements the muscles can perform in a given period of time by isotonic contraction under a specific load. Measurements of muscular endurance during the test consist of three main indicators: weight, time and repetitions. As 60–70% of the weight of the bench press 1RM is the optimal weight for bench press muscular endurance testing, and considering that most of the participants enrolled in this study were non-athletic, a weight of 60% of the 1RM was adopted. Participants were asked to perform the test in one repetition with no pauses in between. To determine the participant’s exhaustion, two criteria were established: (1) the participant paused for more than 2 s after lifting or lowering, or the movement was severely distorted; (2) the participant initiated the release. The final number of repetitions was recorded for each participant [52].

### 2.14. Statistics

All results are presented as mean ± standard deviation (S.D.). The unpaired two-tailed *t*-test was used for two group comparisons, and ordinary one-way ANOVA followed by Dunnett’s post hoc test was used for multiple group comparisons. The testing levels of significance were set at *p* < 0.05 (*), *p* < 0.01 (**) and *p* < 0.001 (***); ns = non-significant. All statistical analyses were performed with Prism 7.0 (GraphPad) and SPSS 22.0 software [53].

## 3. Results

### 3.1. Synergistic Effects of Rhodiola rosea and Caffeine Supplementation on Rats

#### 3.1.1. Forelimb Grip Strength

**Figure 2:**
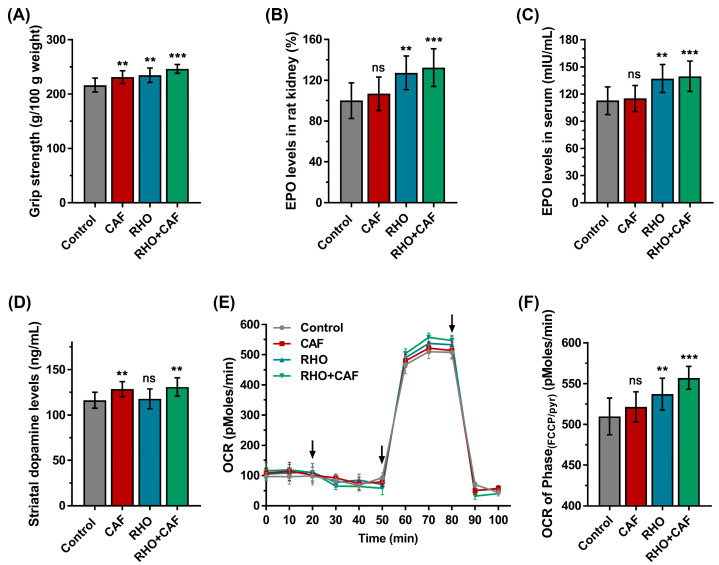
Improvements following supplement treatment in SD rats. (**A**) Forelimb grip strength. (**B**) EPO gene expression in rat kidney. (**C**) Erythropoietin in serum. (**D**) Striatal dopamine. (**E**) Average oxygen consumption rates (OCR, pMoles/min) in single muscle fibers isolated from bilateral gastrocnemius of rats. Arrows represent successive additions of Oligomycin (1 μg/mL), FCCP (400 nM) + pyruvate (10 mM), and Antimycin (1 μM). (**F**) OCR of Phase(FCCP/pyr) in (**E**) for maximal mitochondrial respiration. All data are presented as mean ± SD (n = 12 × 4 groups = 48 samples). ns indicates no statistical difference, ** indicates *p* < 0.01 and *** indicates *p* < 0.001 compared with control group.

To initially assess the synergistic effects of RHO and CAF at the rat level, the forelimb grasp test was used first to measure the effects of placebo, CAF, RHO and CAF+RHO on muscle strength in rats. As shown in Figure 2A, the forelimb grip strength of the placebo group was 216.7 g/100 g body weight. Compared to the placebo group, the forelimb grip strength was increased in the RHO, CAF and RHO+CAF groups. The RHO group reached 234.8 g/100 g body weight (8.35% higher than the placebo group, *p* = 0.0012, partial η^2^ = 0.346), the CAF group reached 231.4 g/100 g body weight (6.78% higher than the placebo group, *p* = 0.0094, partial η^2^ = 0.2832) and the RHO+CAF group reached 246.5 g/100 g body weight (13.75% higher than the placebo group, *p* < 0.0001, partial η^2^ = 0.7141). The results showed that the RHO+CAF group could effectively improve the forelimb grip strength of rats.

#### 3.1.2. EPO Expression Levels in Serum and Rat Kidney

EPO gene expression levels in rats’ kidneys of placebo, RHO, CAF and RHO+CAF groups were measured by qPCR with using the β-actin as the endogenous control (Figure 2B). EPO mRNA levels increased by 27.24% and 32.46% in the RHO (*p* = 0.0010, partial η^2^ = 0.4125) and RHO+CAF groups (*p* = 0.0001, partial η^2^ = 0.4719), respectively, compared to the placebo group. However, the expression of EPO mRNA was not significantly increased in the CAF group (*p* = 0.6488, partial η^2^ = 0.04227) and was only 6.83% higher than that in the placebo group. The above two results suggested that both the RHO and RHO+CAF groups caused an increase in EPO levels.

Additionally, increased serum EPO levels have been shown to improve various physical performances. The EPO ELISA kit was applied here to accurately examine the effect of the RHO+CAF group on serum EPO levels (Figure 2C). Serum EPO levels were significantly higher in the RHO and RHO+CAF treatment groups compared to the placebo group, whose EPO level was 112.8 mIU/mL. RHO given for 30 days without CAF showed an increase in EPO levels to 137.2 mIU/mL (21.63% more than placebo, *p* = 0.0012, partial η^2^ = 0.4048), while RHO given at the same concentration for 30 days and treated with CAF on the last day showed an increase in EPO to 139.7 mIU/mL (23.85% more than placebo, *p* = 0.0003, partial η^2^ = 0.4336). However, CAF administration on the last day alone did not result in a significant increase in EPO, so the level remained almost unchanged at 115.2 mIU/mL (2.13% more than placebo, *p* = 0.9649, partial η^2^ = 0.006998).

#### 3.1.3. Striatal Dopamine Secretion Levels

Dopamine is an important neurotransmitter associated with fatigue, and increasing the level of dopamine is beneficial for improving physical performance [54]. As shown in Figure 2D, dopamine levels in striatal homogenates were higher in the CAF and RHO+CAF treatment groups than that in the placebo group, which had dopamine level of 116.2 ng/mL. CAF given alone on the last day increased the dopamine level to 128.5 ng/mL (10.58% higher than in the placebo group, *p* = 0.0086, partial η^2^ = 0.3585), whereas CAF administered on the last day after 30 days of RHO increased the dopamine level to 130.9 ng/mL (12.65% higher than in the placebo group, *p* = 0.0015, partial η^2^ = 0.3988). The dopamine level in the RHO administration group was 117.8 ng/mL (*p* = 0.9567, partial η^2^ = 0.007377), which was not statistically different from the placebo group. The above two results indicate that both the CAF and the RHO+CAF groups caused an increase in dopamine levels.

#### 3.1.4. Oxygen Consumption Rate Measurement of Muscle Fibers

Exercise-induced muscle contraction consumes energy, which is provided by oxidative phosphorylation of mitochondria. Therefore, individual muscle fibers of the bilateral gastrocnemius muscle of male SD rats were isolated to examine the mitochondrial respiration. As shown in Figure 2E, there were no obvious changes in OCR in Phase(Oligo) following Oligomycin addition between the four groups. However, after stimulation by membrane-permeable FCCP as the uncoupler, all the administrations of CAF, RHO and RHO+CAF enhanced the mitochondrial respiration to some extent in Phase(FCCP/pyr) compared to the placebo group (Figure 2F). Among them, RHO+CAF most efficiently improved the maximal OCR than other groups (*p* ˂ 0.05), indicating the synergistic effect of RHO and CAF on improving the capacity of mitochondrial aerobic respiratory.

### 3.2. Daily Recordings of Carbohydrate, Protein, Fat and Energy in Dietary Intake

**Figure 3:**
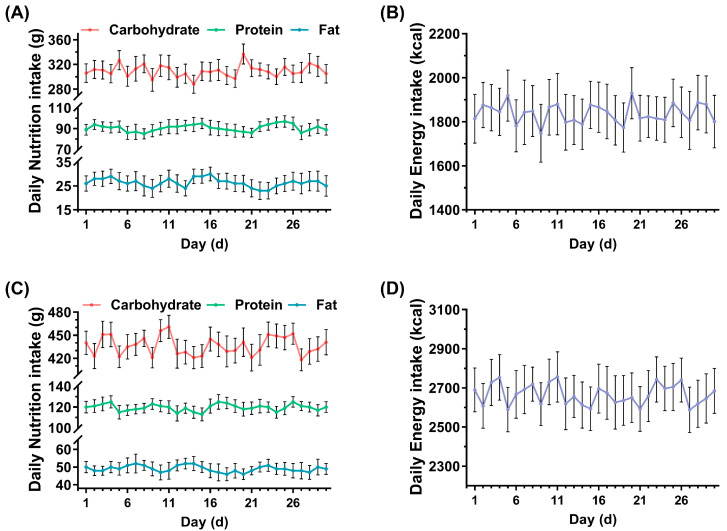
Daily records of carbohydrate, protein, fat and energy in dietary intake. Daily nutrition intake (**A**) and energy intake (**B**) of resistance exercise-untrained volunteers (n = 48). Daily nutrition intake (**C**) and energy intake (**D**) of resistance exercise-trained volunteers (n = 24).

In order to avoid the possible impact from the dietary factors, several fixed packages were given to each resistance exercise-untrained and -trained volunteer to choose from. The dietary intakes of resistance exercise-untrained volunteers were approximately 310 g of carbohydrates, approximately 91 g of protein and approximately 26 g of fat (Figure 3A), and the total energy intake was approximately 1838 kcal (Figure 3B). In comparison, the dietary intakes of resistance exercise-trained volunteers were approximately 437 g of carbohydrates, approximately 120 g of protein and approximately 49 g of fat (Figure 3C), and the total energy intake was approximately 2666 kcal (Figure 3D). All participants maintained their habitual diets throughout the study.

### 3.3. Changes for Participants without Resistance Training Experience after Receiving Supplements with Concomitant Training

#### 3.3.1. Bench Press 1RM and Deep Squat 1RM

**Figure 4:**
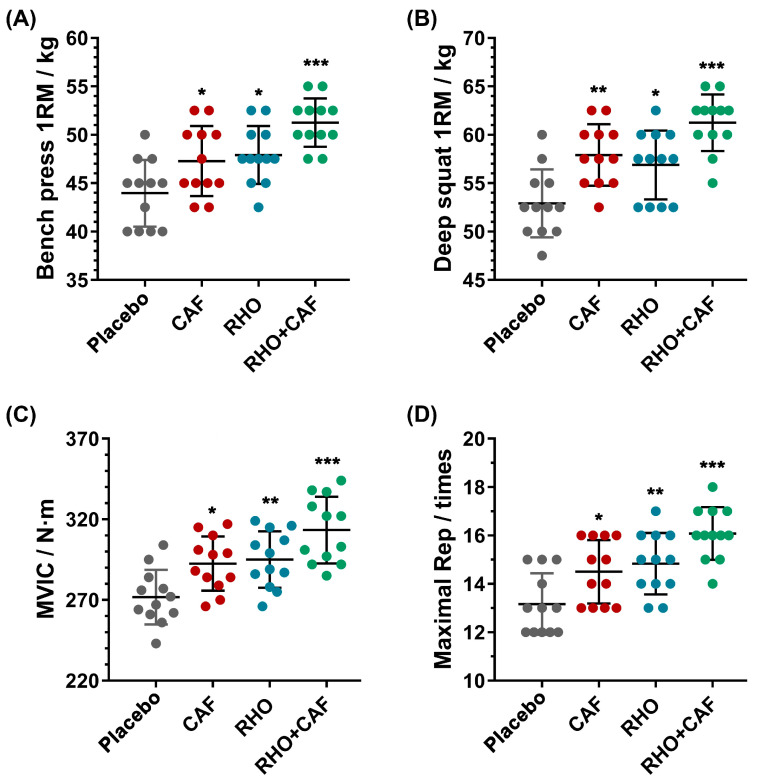
Changes for participants without resistance training experience after receiving supplements. (**A**) Bench press 1RM. (**B**) Deep squat 1RM. (**C**) MVIC. (**D**) Maximal repetitions of 60% 1RM bench press. All data are presented as mean ± SD (n = 12 × 4 groups = 48 samples). * indicates *p* < 0.05, ** indicates *p* < 0.01 and *** indicates *p* < 0.001 compared with placebo group.

The initial bench press 1RM values for the recruited groups were 25.21 ± 3.10 kg (placebo), 24.79 ± 3.45 kg (CAF), 24.58 ± 3.34 kg (RHO) and 25.00 ± 3.02 kg (CAF+RHO) respectively, while the initial deep squat 1RM values were 36.25 ± 4.20 kg (placebo), 36.04 ± 3.76 kg (CAF), 35.83 ± 4.17 kg (RHO) and 35.63 ± 4.28 kg (CAF+RHO). There were no significant differences between the initial values of the groups, thus allowing for the follow-up study. As shown in Figure 4A,B, the placebo group had a bench press 1RM of 43.96 kg and a deep squat 1RM of 52.92 kg. Compared to the placebo group, the CAF, RHO and RHO+CAF groups all showed improvements in both bench press 1RM and deep squat 1RM. Specifically, for the bench press 1RM, the CAF group increased to 47.29 kg (7.58% higher than the placebo group, *p* = 0.0353, partial η^2^ = 0.196), the RHO group increased to 47.92 kg (9.00% higher than the placebo group, *p* = 0.0103, partial η^2^ = 0.2956) and the CAF and RHO combination group increased to 51.25 kg (16.59% higher than the placebo group, *p* < 0.0001, partial η^2^ = 0.6368). For the deep squat 1RM, the CAF group increased to 57.92 kg (9.45% higher than the placebo group, *p* = 0.0016, partial η^2^ = 0.3814), the RHO group increased to 56.88 kg (7.48% higher than the placebo group, *p* = 0.0143, partial η^2^ = 0.2552) and the combined CAF and RHO group increased to 61.25 kg (15.75% higher than the placebo group, *p* < 0.0001, partial η^2^ = 0.6526). Both the 30-day RHO and one-time high-dose CAF improved the bench press 1RM and deep squat 1RM to varying degrees compared to the placebo group, respectively, and the 30-day RHO combined with the one-time high-dose CAF further maximized the physical performance.

#### 3.3.2. MVIC and Maximal Repetitions of 60% 1RM Bench Press

The initial MVIC values for the recruited groups were 179.17 ± 14.50 kg (placebo), 180.25 ± 13.43 kg (CAF), 177.42 ± 14.29 kg (RHO) and 178.67 ± 13.13 kg (CAF+RHO), while the maximal repetitions of 60% 1RM bench press values were 10.42 ± 1.08 kg (placebo), 10.25 ± 0.97 kg (CAF), 10.33 ± 1.07 kg (RHO) and 10.17 ± 1.11 kg (CAF+RHO). It is clear that there were no significant differences between the initial values of the groups, and therefore, a follow-up study could be carried out. As shown in Figure 4C,D, the MVIC in the placebo group was 271.75 N∙m, while the maximal repetitions of 60% 1RM bench press amounted to 13.17 times. The CAF, RHO and RHO+CAF groups all increased MVIC and maximal repetitions of 60% 1RM bench press compared to the placebo group. In particular, for MVIC, the CAF group increased to 292.58 N∙m (7.67% higher than the placebo group, *p* = 0.0191, partial η^2^ = 0.2939), the RHO group increased to 295.08 N∙m (8.59% higher than the placebo group, *p* = 0.0078, partial η^2^ = 0.3343) and the CAF and RHO combination group increased to 311.75 N∙m (14.72% higher than the placebo group, *p* < 0.0001, partial η^2^ = 0.5796). For the maximal repetitions of 60% 1RM bench press, the CAF group increased to 14.50 times (10.01% higher than the placebo group, *p* = 0.0304, partial η^2^ = 0.2256), the RHO group increased to 14.83 times (12.66% higher than the placebo group, *p* = 0.0054, partial η^2^ = 0.3205) and the combined CAF and RHO group increased to 16.08 times (22.15% higher than the placebo group, *p* < 0.0001, partial η^2^ = 0.6309). The RHO group and CAF group both improved the MVIC and maximal repetitions of 60% 1RM bench press to different degrees, respectively.

### 3.4. Improvements following Supplement Treatment for Resistance Exercise-Trained Volunteers

**Figure 5:**
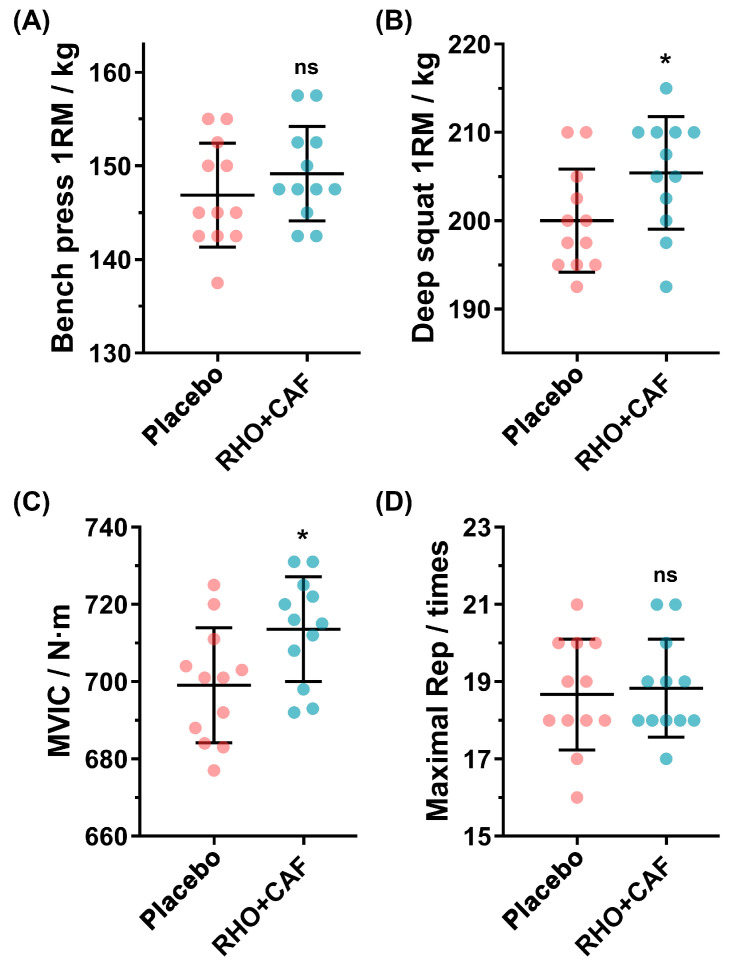
Improvements following supplement treatment for resistance exercise-trained volunteers. (**A**) Bench press 1RM. (**B**) Deep squat 1RM. (**C**) MVIC. (**D**) Maximal repetitions of 60% 1RM bench press. All data are presented as mean ± SD (n = 12 × 2 groups = 24 samples). ns indicates no statistical difference and * indicates *p* < 0.05 between the compared groups.

The initial bench press 1RM values for the recruited resistance exercise-trained volunteers were 139.58 ± 3.17 kg (placebo) and 139.79 ± 3.61 kg (CAF+RHO), while the initial deep squat 1RM values were 192.08 ± 3.82 kg (placebo) and 191.67 ± 3.59 kg (CAF+RHO). Meanwhile, the initial MVIC values were 684.08 ± 10.85 kg (placebo) and 682.58 ± 11.33 kg (CAF+RHO), while the maximal repetitions of 60% 1RM bench press values were 17.92 ± 1.16 kg (placebo) and 18.17 ± 0.83 kg (CAF+RHO). It was evident that there was not a significant difference between the groups’ initial values, and therefore, a subsequent investigation could be conducted. All the above-demonstrated results suggest that 30 days of dosing of RHO combined with acute CAF supplementation can better enhance the physical performance of volunteers without resistance training experience compared to RHO or CAF alone. Based on these encouraging results, the effectiveness of the current prescription (placebo and RHO+CAF) was further investigated in resistance exercise-trained volunteers with 5.7 ± 0.7 years of resistance training experience to test their capabilities of improving physical performance. As shown in Figure 5, 12 resistance exercise-trained volunteers after administration of RHO+CAF with routine training showed practical improvements in all metrics, including bench press 1RM, deep squat 1RM, MVIC and maximal repetitions of 60% 1RM bench press; meanwhile, deep squat 1RM and MVIC had statistically significant differences.

## 4. Discussion

The main finding of this study was that 30 days of dosing of RHO combined with acute doses of CAF had a significant boosting effect on both rat models (including forelimb grip strength, EPO expression levels in rat kidney and serum, striatal dopamine secretion level and oxygen consumption rate) and resistance exercise-untrained volunteers (including classic strength indicators such as bench press 1RM, deep squat 1RM, MVIC and maximal repetitions of 60% 1RM bench press). This supplementation is not only suitable for people with no previous exercise experience, but also suitable for these resistance exercise-trained volunteers, which is consistent with the ability of CAF and RHO to improve physical performance [7,55].

To investigate the synergistic effects of RHO and CAF supplementation on improving muscle strength and muscle endurance, we first selected SD rats as the study model. The conventional forelimb grip strength test is a widely used non-invasive method for assessing skeletal muscle function in rats. Rats were given appropriate doses of RHO and CAF during 30 days of classical weighted climbing resistance training, and the results showed that the RHO+CAF group was able to improve forelimb grip strength by 18.0% compared to the placebo group. Previous studies have suggested that the role of RHO in improving muscle strength and muscle endurance may be related to its capability to increase EPO expression [4]. Therefore, this study investigated both EPO gene expression in rat kidneys (gene level) and serum EPO levels (protein level), showing that EPO gene expression increased by 32.46% after RHO+CAF treatment compared to the placebo, while its serum EPO levels also increased by 23.85% compared to the placebo. These results suggest that the RHO+CAF group can effectively upregulate EPO expression, which is one of the most important characteristics to improve physical performance. Similar to the present results, several studies on RHO have shown that it can improve hematopoiesis by increasing EPO mRNA expression through activation of the PI3K/Akt/HIF-1α signaling pathway. RHO has also been shown to reduce IL-6, IL-1β, TNF-α, CD14, CD44 and iNOs mRNA and improve hematopoiesis by upregulating the protective proteins thioredoxin-1 and glutathione peroxidase-1, with an overall positive effect on consumers. While these studies support our contention that RHO can promote EPO expression, they also inspire us that there may be other potentially relevant signaling pathways for RHO to promote exercise that merit further investigation [4,56,57].

Moreover, CAF could effectively promote the release of dopamine and other neurotransmitters [17], thus further delaying fatigue and improving athletic performance. In recent years, caffeine above 3 mg/kg has been shown to be very effective in improving various exercise capacities. We measured striatal dopamine levels in each group, and the results showed a 12.65% increase in dopamine levels in the RHO+CAF compared to the placebo group, which may be responsible for the improved physical performance through reduced fatigue. In recent years, many studies have shown that CAF has a proven potentiating effect on athletic performance, which is reproducible and shows positive effects in a variety of different sports (including muscular endurance, movement velocity and muscular strength, sprinting, jumping, and throwing performance, as well as a wide range of aerobic and anaerobic sport-specific actions) and is therefore used by many athletes and non-athletes. Although the exact mechanism of action is unknown, a growing body of research has shown that caffeine substitutes, such as caffeine-containing mouthwashes, chewing gums, energy gums and chewable tablets, have been shown to be effective in improving athletic performance, which also suggests a link to dopamine. In the present study, a positive correlation between CAF and dopamine was also shown, as well as a positive correlation with exercise facilitation capacity, although a complete mechanism of correlation was not demonstrated [58,59,60].

Oxygen consumption rate is a critical indicator for assessing mitochondrial respiration in skeletal muscle, which is the largest contributor to the aerobic capacity of the whole body [61]. Maximal oxygen consumption of muscle fibers enhanced by RHO+CAF indicates a corresponding increase in the activity of mitochondrial respiration for oxidative phosphorylation, which in turn leads to an enhancement in physical performance. Overall, animal studies have demonstrated from several perspectives that RHO+CAF produces better effects and can potentially work on the improvement of muscle strength and muscle endurance than using RHO or CAF alone at multiple levels. Similar to the findings in this study, other studies have shown that caffeine increases V_O2max_ in elite athletes, which contributes to improved high-intensity endurance performance. Increases in O_2_ deficit and lactate also contributed to caffeine-induced improvements in endurance performance [62]. Similarly, studies on RHO have shown that RHO can significantly improve a number of metrics such as peak V_O2_, time to exhaustion, peak power output and peak heart rate, all of which support our findings to some extent, and we hope to investigate these metrics more in future studies [63].

We then recruited resistance exercise-untrained volunteers to revalidate the effect of RHO+CAF on the human level, and the results showed that this group was more effective in increasing the bench press 1RM, deep squat 1RM, MVIC and maximal repetitions of 60% 1RM bench press compared to RHO or CAF group alone.

The bench press and deep squat 1RM are the most obvious and classic indicators of absolute strength, while the MVIC and maximal repetitions of 60% 1RM bench press are essential indicators for isotonic muscle strength and endurance. In this study, 30 days of dosing of RHO improved 9.00% in bench press 1RM, 7.48% in deep squat 1RM, 12.66% in maximal repetitions of 60% 1RM bench press and 8.59% in MVIC compared to the placebo group. The maximal repetitions of 60% 1RM bench press showed stronger changes compared to the bench press 1RM, deep squat 1RM and MVIC. All these enhancements in the skeletal muscle capacities are reasonably inferred to as the potential improvement of the contents and functions of hemoglobin by RHO, i.e., the RHO-induced hemoglobin production facilitated by EPO can further increase the oxygen-carrying and oxygen-supplying capacity of the blood to ultimately improve exercise performance [64]. Considering that the average number of repetitions is around 20, it is not known whether the oxidative capacity or performance of EPO is important in the repetition test of resistance training, and this remains to be investigated. In our rat model (Figure 2B,C), RHO has been verified to effectively increase the transcription and translation of EPO, thereby enhancing the sustained upregulation of hemoglobin counts in vivo [65]. Similarly, a large number of studies based on both male and female subjects have shown that acute CAF supplementation can be effective in improving bench press 1RM and deep squat 1RM, and although the details of these studies vary, the general trend is the same [66,67]. Meanwhile, RHO was effective in improving the subjects’ bench press 1RM and deep squat 1RM, results that are consistent with the results of this study [68].

In this study, CAF improved 7.58% in bench press 1RM, 9.45% in deep squat 1RM, 10.01% in maximal repetitions of 60% 1RM bench press and 7.67% in MVIC compared to the placebo group. This result is supported by multiple shreds of evidence that both the upper body strength and lower body strength were improved by the doses of CAF, verified by a significant effect on the bench press 1RM and deep squat 1RM [69]. CAF has been shown to produce greater improvements in knee extensor strength, as knee extensors tend to be activated at lower levels than upper body muscle groups during maximal voluntary contraction. Therefore, the deep squat 1RM produced a more significant improvement than the bench press 1RM, which could explain the higher effect found in our results for the deep squat 1RM compared to the bench press 1RM [70]. Meanwhile, after taking a dose of CAF, the adenosine receptors in the body are rapidly activated and a variety of stimulant neurotransmitters (dopamine, norepinephrine and serotonin) in the central and peripheral nervous systems are rapidly released into action, which further improves exercise performance [18]. Our animal experiments also demonstrated a significant increase in striatal dopamine levels after CAF administration (Figure 2D), while dopamine elevated by CAF can reveal an anti-fatigue effect. Many related studies have also shown that CAF can help restore impaired MVIC in patients with delayed-onset muscle soreness after exercise-induced muscle damage, as well as restore the decline in MVIC in naturally menstruating women during the early follicular phase of resistance training and reduce delayed-onset muscle pain and loss of MVIC after eccentric exercise, findings that partly support the results of our study [71,72,73].

Consuming a combination of RHO and CAF can improve the performance of male recreational exercisers in the 5 km running time trial [74]. Therefore, in this study, we identified the combination of CAF+RHO can produce better physical performance-boosting effects than either alone, and we further investigated the potential mechanisms for this process.

The combination of these two different supplements also mechanistically resulted in a greater enhancement of exercise capacity, as expected. In this study, a 16.59% increase in bench press 1RM, a 15.75% increase in deep squat 1RM, a 22.15%% increase in maximal repetitions of 60% 1RM bench press and a 14.72%% increase in MVIC were found in the combined dosing groups. These results also indicated that the CAF+RHO group can produce better physical performance improvement than using CAF or RHO alone. This may be due to the fact that a single intake of RHO can increase the amount of hemoglobin by the upregulated EPO (as shown in Figure 2B,C), but the body was unable to mobilize the muscles effectively for the short burst of force to maximize the effect. With the combination of the dose of CAF, the organism was not only able to build on the significant increase in hemoglobin brought by RHO, but also to further increase the attention to force and maintain a state of arousal and reduce fatigue by CAF-induced dopamine (as shown in Figure 2D), improving the neural recruitment capacity of the muscles and ultimately achieve a stronger facilitation effect than the other groups.

The above results suggest that RHO+CAF was effective in improving the physical performance of volunteers without resistance training experience. Resistance exercise-trained volunteers with years of resistance training experience also need nutritional supplements to boost physical performance and push their limits. A total of 24 resistance exercise-trained volunteers with relevant experience (5.7 ± 0.7 years) were recruited to validate the effect on physical performance by combined administration of RHO+CAF. Overall, the bench press 1RM, deep squat 1RM, MVIC and maximal repetitions of 60% 1RM bench press showed an increasing trend, while the deep squat 1RM and MVIC showed statistical differences in this increase. Although bench press 1RM and maximal repetitions of 60% 1RM bench press had no statistical difference compared with the placebo group, a trend towards improvement was evident. This may be due to the fact that after a long period of training, resistance exercise-trained volunteers are close to their limits and have a “ceiling” effect, which does not easily produce significant improvements. In addition, contrary to our results, the acute ergogenic effect of caffeine on resistance exercise performance was not moderated by training status [75].

## 5. Conclusions

In summary, the present study demonstrates that a 30-day supplementation of RHO combined with a single dose of CAF seems to improve the performance of resistance exercises in resistance exercise-untrained volunteers. Meanwhile, forelimb grip strength was enhanced in rats given the corresponding simulated dose of RHO+CAF. The increased levels of EPO, dopamine and maximal oxygen consumption in the rat model also provide certain evidence of the potential mechanisms for enhanced exercise capacities associated with the combined use of RHO and CAF. For resistance exercise-trained volunteers, RHO+CAF may also have a positive effect, contributing to pushing their limits further.

## Figures and Tables

**Table 1:** The somatotype profile of the resistance exercise-untrained volunteers and resistance exercise-trained volunteers.

	Resistance Exercise-Untrained Volunteers (n = 48)	Resistance Exercise-Trained Volunteers (n = 24)
Age/year	20.5 ± 2.6	23.6 ± 2.2
Body height/cm	175.2 ± 5.1	178.5 ± 4.2
Body weight/kg	75.1 ± 7.0	87.5 ± 5.9
BMI/kg·m^−2^	24.37 ± 1.72	27.42 ± 1.12
Wrist diameter/cm	6.26 ± 0.82	6.79 ± 0.67
Elbow diameter/cm	7.01 ± 0.94	7.70 ± 0.81
Knee diameter/cm	9.12 ± 1.02	9.18 ± 0.54
Ankle diameter/cm	7.26 ± 1.04	7.48 ± 0.75
Circumference of thigh/cm	53.7 ± 3.31	62.5 ± 4.83
Circumference of calf/cm	39.4 ± 2.67	42.5 ± 2.84
Circumference of forearm/cm	33.5 ± 2.47	33.9± 2.31
Circumference of relaxed upper arm/cm	34.7 ± 3.10	38.6 ± 3.30
Skinfold thickness of thigh-ventral/mm	7.75 ± 2.55	7.14 ± 3.06
Skinfold thickness of calf/mm	5.22 ± 1.93	5.19 ± 2.27
Skinfold thickness of biceps brachii/mm	3.65 ± 1.28	3.06 ± 1.34
Skinfold thickness of triceps brachii/mm	5.77 ± 2.09	4.32 ± 1.46
Skinfold thickness of forearm-volar/mm	3.80 ± 1.46	3.64 ± 1.50

## Data Availability

The original contributions presented in the study are included in the article; further inquiries can be directed to the corresponding author.
